# Automatic anatomical foot and ankle coordinate toolbox

**DOI:** 10.3389/fbioe.2023.1255464

**Published:** 2023-10-31

**Authors:** Andrew C. Peterson, Karen M. Kruger, Amy L. Lenz

**Affiliations:** ^1^ Department of Orthopaedics, University of Utah, Salt Lake City, UT, United States; ^2^ Department of Biomedical Engineering, Marquette University, Milwaukee, WI, United States; ^3^ Motion Analysis Center, Shriners Children’s, Chicago, IL, United States; ^4^ Department of Biomedical Engineering, University of Utah, Salt Lake City, UT, United States

**Keywords:** automated toolbox, foot and ankle, coordinate system, anatomic, morphological variation, foot deformities

## Abstract

Accurate analysis of bone position and orientation in foot and ankle studies relies on anatomical coordinate systems (ACS). Reliable ACSs are necessary for many biomechanical and clinical studies, especially those including weightbearing computed tomography and biplane fluoroscopy. Existing ACS approaches suffer from limitations such as manual input, oversimplifications, or non-physiological methods. To address these shortcomings, we introduce the Automatic Anatomical Foot and Ankle Coordinate Toolbox (AAFACT), a MATLAB-based toolbox that automates the calculation of ACSs for the major fourteen foot and ankle bones. In this manuscript, we present the development and evaluation of AAFACT, aiming to provide a standardized coordinate system toolbox for foot and ankle studies. The AAFACT was evaluated using a dataset of fifty-six models from seven pathological groups: asymptomatic, osteoarthritis, pilon fracture, progressive collapsing foot deformity, clubfoot, Charcot Marie Tooth, and cavovarus. Three analyses were conducted to assess the reliability of AAFACT. Firstly, ACSs were compared between automatically and manually segmented bone models to assess consistency. Secondly, ACSs were compared between individual bones and group mean bones to assess within-population precision. Lastly, ACSs were compared between the overall mean bone and group mean bones to assess the overall accuracy of anatomical representation. Statistical analyses, including statistical shape modeling, were performed to evaluate the reliability, accuracy, and precision of AAFACT. The comparison between automatically and manually segmented bone models showed consistency between the calculated ACSs. Additionally, the comparison between individual bones and group mean bones, as well as the comparison between the overall mean bone and group mean bones, revealed accurate and precise ACSs calculations. The AAFACT offers a practical and reliable solution for foot and ankle studies in clinical and engineering settings. It accommodates various foot and ankle pathologies while accounting for bone morphology and orientation. The automated calculation of ACSs eliminates the limitations associated with manual input and non-physiological methods. The evaluation results demonstrate the robustness and consistency of AAFACT, making it a valuable tool for researchers and clinicians. The standardized coordinate system provided by AAFACT enhances comparability between studies and facilitates advancements in foot and ankle research.

## 1 Introduction

Anatomical coordinate systems (ACS) play a critical role in analyzing bone position and orientation during various static and dynamic movements. These coordinate systems are utilized for measuring changes in ankle joint angles during gait (Canton et al., 2020), comparing the kinematics of healthy individuals to those with malformed foot and ankle bones (Lenz et al., 2020; Lenz et al., 2022), and assessing the success of surgical procedures when correcting different pathological deformities (Taniguchi et al., 2021). It is essential to define an accurate ACS that captures the unique morphology and alignment of the bones, as it ensures precise measurement of joint angles and assessment of deformity correction. Additionally, to appropriately compare findings between studies, a widely accepted definition for individual bones is needed (Wu et al., 2002). Currently, there are many foot and ankle coordinate system approaches. Some methods involve manually identifying bony landmarks (Liu et al., 2007; Yamaguchi et al., 2009; Ito et al., 2015; Claassen et al., 2019) or fitting surfaces to geometric primitives (de Asla et al., 2006; Yamaguchi et al., 2009; Green et al., 2011), while others apply the coordinate system of one bone for all subsequent bones (Wang et al., 2015; Roach et al., 2016), or rely on kinematic mathematical methods (Grood and Suntay, 1983; Woltring et al., 1994; Ledoux et al., 2006). These various approaches have their limitations, such as manual input, oversimplifications, or reliance on non-physiological mathematical methods. Therefore, a pressing need exists for an automatic and reliable coordinate system toolbox that can overcome these limitations and provide accurate coordinates for individual bones in the foot and ankle (Knutson et al., 2023).

In this study, we present an open-source toolbox developed to calculate the ACSs automatically for each of the fourteen foot and ankle bones. This toolbox will be referred to as Automatic Anatomical Foot and Ankle Coordinate Toolbox, or AAFACT. We tested the toolbox on fifty-six models, eight individuals in seven different pathological categories: asymptomatic, osteoarthritis (OA), pilon fracture, progressive collapsing foot deformity (PCFD), clubfoot, Charcot-Marie-Tooth (CMT), and cavovarus. To assess the dependability of the toolbox, we conducted three analyses: comparing ACSs from automated vs manual bone segmentations, individual vs group mean bone ACSs, and overall vs group mean bone ACSs. These analyses were performed to assess the toolbox’s effectiveness in various populations exhibiting a range of deformities.

This manuscript aims to report the development of our AAFACT definition for each of the fourteen foot and ankle bones and to evaluate the consistency of the coordinate definitions across different segmentation processes and populations with various deformities. We aim to develop a practical and reliable coordinate system toolbox that can be easily used in clinical or engineering settings and account for various spectrums of foot and ankle pathologies, regardless of bone morphology or orientation.

## 2 Materials and methods

### 2.1 Image acquisition and processing

Fifty-six participants were retrospectively used for this study. Individuals who received a weightbearing computed tomography (WBCT) scan with a full field of view were included. A full field of view was achieved if the twelve bones distal to the tibia and fibula were fully present in the scan. These bones included the talus, calcaneus, navicular, cuboid, medial cuneiform, intermediate cuneiform, lateral cuneiform, and all five metatarsals. Only a distal field of view for the tibia and fibula bones were included. Of the fifty-six participants, pathologically, eight were asymptomatic (CurveBeam PedCAT; 0.37 mm³ voxel size), eight had end-stage tibiotalar and subtalar OA (CurveBeam PedCAT; 0.37 mm³ voxel size), eight were recovering from a pilon fracture reconstruction (CurveBeam PedCAT; 0.37 mm³ voxel size), eight were diagnosed with PCFD (Planmed Verify, Planmed Oy; 0.4 mm³ voxel size), eight were diagnosed with clubfoot (CurveBeam PedCAT; 0.37 mm³ voxel size), eight were diagnosed with CMT (CurveBeam PedCAT; 0.37 mm³ voxel size), and eight were diagnosed with cavovarus feet (CurveBeam PedCAT; 0.37 mm³ voxel size and Siemens Healthineers Multitom Rax; 0.35 mm³ voxel size). The fifty-six patients were a population of convenience with equal sample size per pathology. Within a population, patients were selected at random to not include selection bias or only severe cases within the pathology. The selected populations were chosen to represent both mild and severe pathologies. Severity here relates to bony anatomy that has either exhibited shape change with respect to healthy bone shape or in a clinical case where patients have pain and limited function. There were no other inclusion criteria for this study. Each WBCT scan was automatically segmented using DISIOR (DISIOR Bonelogic Ortho Foot and Ankle 2.1.1, Helsinki, Finland) and manually cleaned up by a single segmenter with over 1,000 h of experience in foot and ankle segmentations using Mimics (Mimics 24.0; Materialise) to prepare the bone models for analysis. These manually cleaned up bone models will be the gold-standard models for each analysis in this study.

### 2.2 Toolbox development

#### 2.2.1 Toolbox overview

**FIGURE 1:**
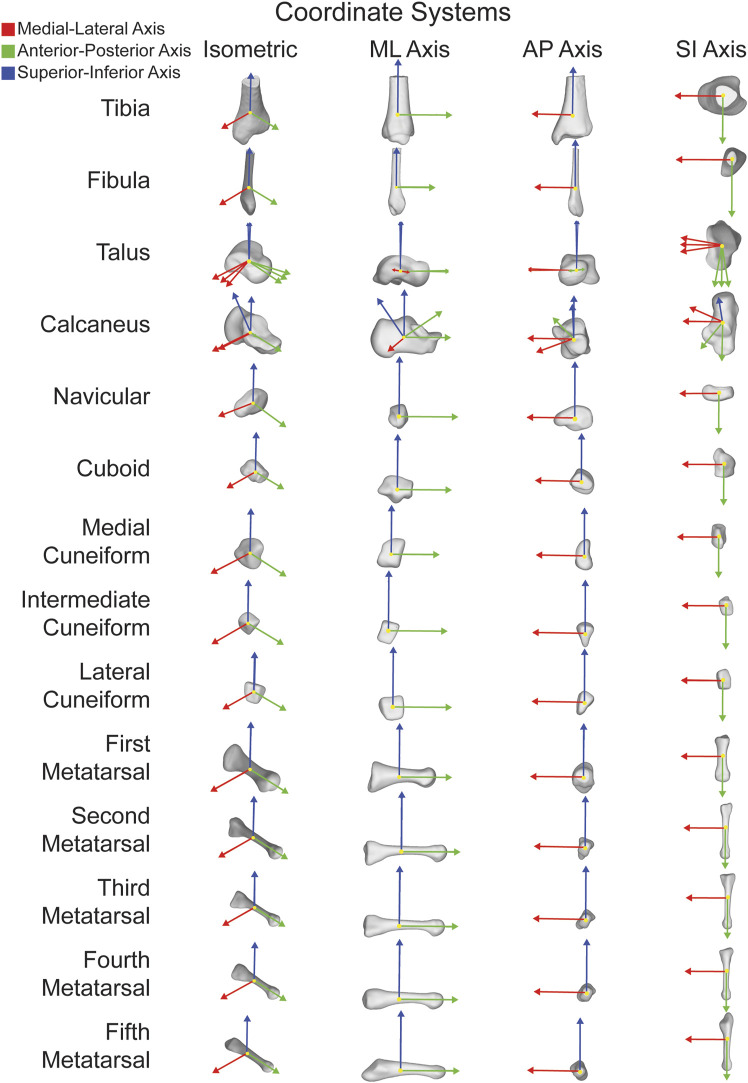
ACSs for fourteen major bones individually shown in isometric views as well as along each orthogonal axes. ML axis shown in red, AP axis shown in green, and SI axis shown in blue.

**FIGURE 2:**
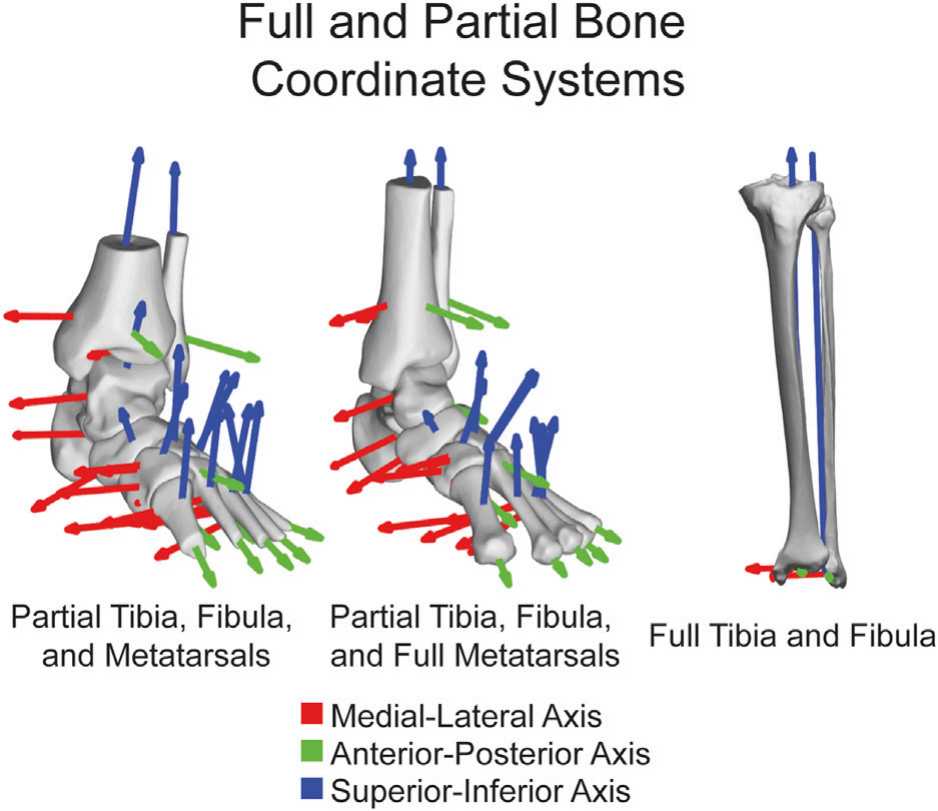
ACSs shown on fourteen major bones together highlighting the functionality on both partial and full tibias, fibulas, and metatarsals. ML axis shown in red, AP axis shown in green, and SI axis shown in blue.

**FIGURE 3:**
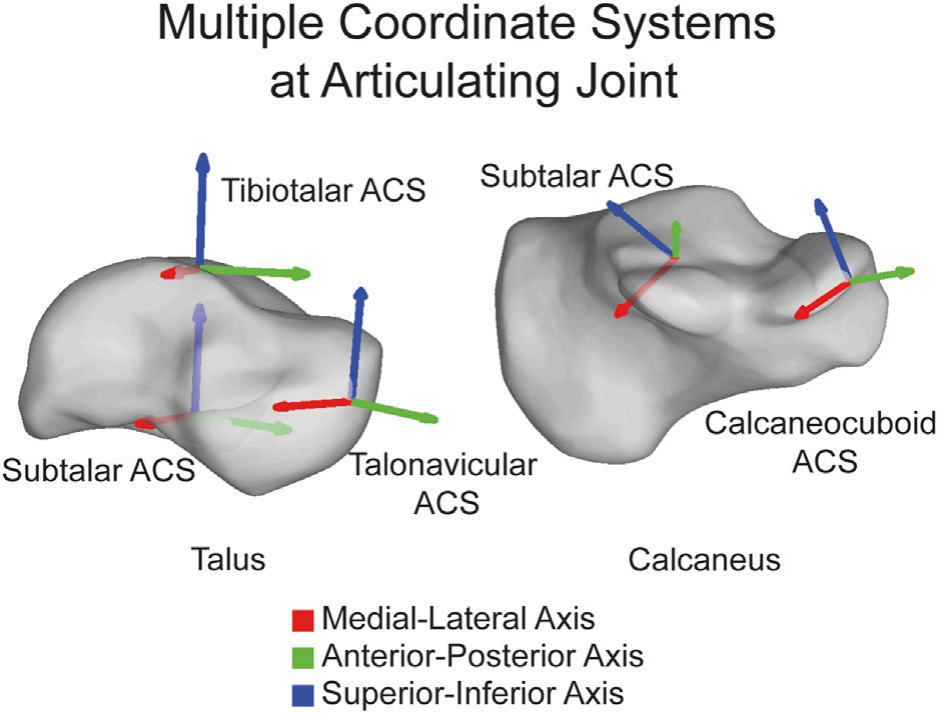
Multiple ACSs shown on the talus and calcaneus highlighting that ACSs can be joint specific and placed directly on the joint. ML axis shown in red, AP axis shown in green, and SI axis shown in blue.

The open-source AAFACT, https://github.com/Lenz-Lab/AAFACT (Peterson, 2023), was developed in MATLAB (R2022b, MathWorks, Natick, MA, United States) to automate the process of assigning coordinates to each of the 14 bones that make up the foot and ankle (Figure 1). The toolbox provides a user-friendly interface, allowing users to load any bone model from the 14 bones in any position and orientation, as long as 3D reconstructions are generated from a comparable native image resolution. Baseline knowledge of foot and ankle anatomy is required for users to generate 3D bone reconstructions from image data within our noted acquisition resolution. The toolbox interface prompts the user to select the folder containing all bones that the user desires to have an ACS applied to. Currently supported file types are: .stl, .k, .particles, .vtk, and .ply. Based on the file name or user input, it automatically determines the bone and laterality, mirroring rights to become left. Due to field of view differences in clinical scanning systems, the AAFACT accommodates full length and partial length tibias, fibulas, and metatarsals (Figure 2). For some bones, such as the calcaneus and talus, the toolbox prompts users to select which anatomical coordinate systems (ACS) they want to define, such as talonavicular, tibiotalar (de Asla et al., 2006; Yamaguchi et al., 2009; Stolle et al., 2022), subtalar (Conconi et al., 2021), or calcaneocuboid. Additionally, the toolbox prompts the user to specify the origin of the ACS, which can be located at the center of the bone or a joint surface (Tuszynski, 2023) (Figure 3).

#### 2.2.2 ACS assignment

Once the bone is loaded and the necessary information is provided, the AAFACT employs an iterative closest point approximation technique to temporarily align the user’s bone to a template bone (Wilm, 2023). The individual template bones for alignment are each overall mean shape developed from a statistical shape model that included all of the deformities in this study encompassing variations in deformity from cavovarus to planovalgus. The templates are pre-oriented with the medial region in the positive *x* direction, the anterior region in the positive *y* direction, and the superior direction in the positive *z* direction. This alignment process ensures consistency in coordinate calculations. For bones that have multiple ACSs, such as the talus and calcaneus, the template bones have slightly different orientations to appropriately target the joints of interest.

**FIGURE 4:**
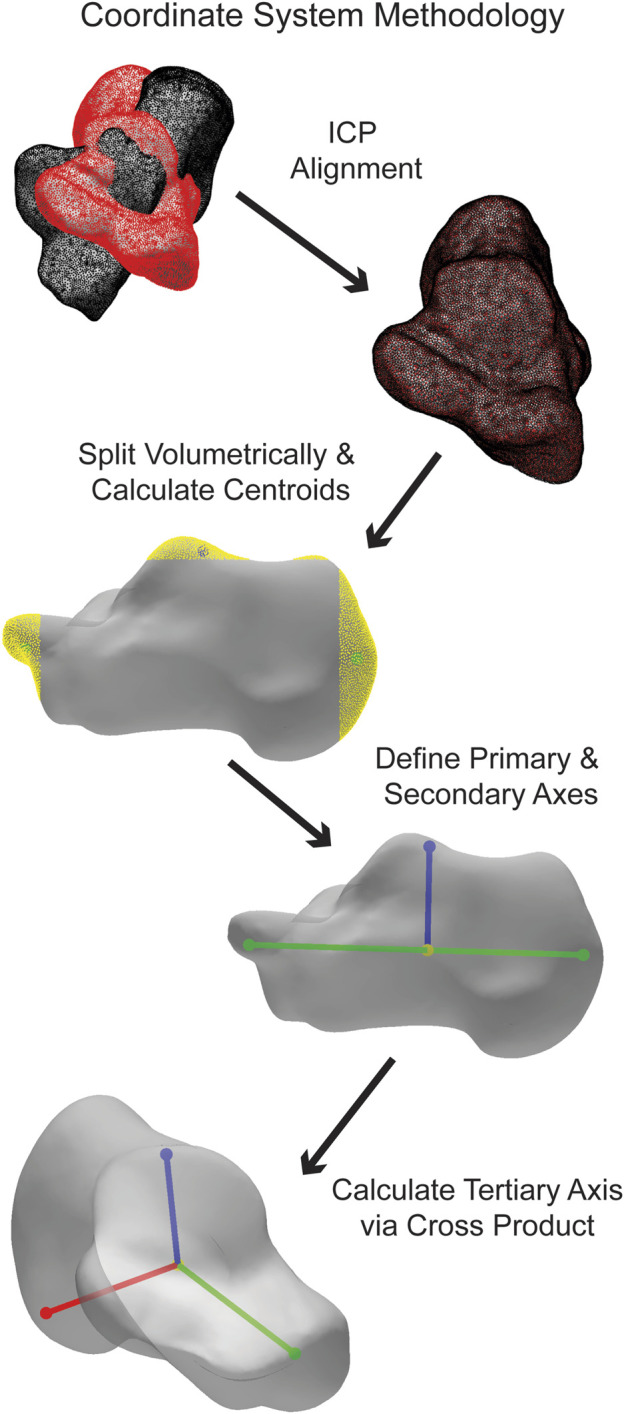
Simplified visualization of steps taken in the AAFACT when assigning an ACS to a calcaneus.

The coordinate calculation details vary slightly for each bone. Generally, the bones are divided into regions in all three planes. The talus, cuneiforms, metatarsals, tibia, and fibula are split into three volumetric regions per plane, the navicular and cuboid into five volumetric regions per plane, and the calcaneus into ten volumetric regions per plane. By defining the centroids of specific regions depending on the bone, the primary axis is formed. The most medial and lateral regions are used for the navicular and tibiotalar talus ACS, and the most superior and inferior regions are used for the tibia and fibula. All other bones use the most anterior and posterior regions for their primary axis. Additionally, a third point is defined as a centroid in a specific region, which varies depending on the bone. The tibia and fibula use the most lateral region, while for all other bones, it is the superior region. An orthogonal line is formed between the third point and the primary axis, forming the secondary axis. Finally, the cross-product between the primary and secondary axes forms the tertiary axis. The three calculated axes correspond to the medial-lateral (ML), anterior-posterior (AP), and superior-inferior (SI) axes. (Figure 4).

#### 2.2.3 Toolbox outputs

The AAFACT provides two primary outputs. First, it generates a visual representation of the ACS overlaid on the bone model. This visual aid helps users visualize and assess the coordinate system (Johnson, 2023). And second, the toolbox produces a spreadsheet that contains the three vectors defining the ACS in both the bone’s original coordinate space and the temporarily aligned space with a local origin applied.

### 2.3 Statistical analysis

#### 2.3.1 Statistical shape model

**FIGURE 5:**
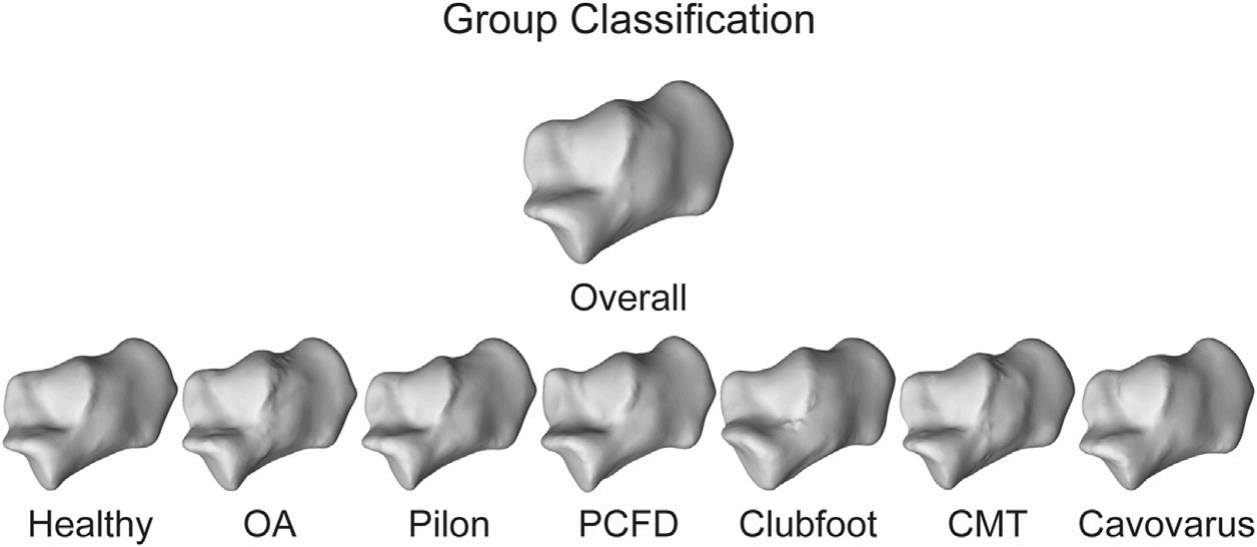
Overall mean shape and the mean shapes of all the groups for the calcaneus to represent anatomical variation.

Three different approaches were taken to statistically determine the toolbox’s consistency, accuracy, and precision. A statistical shape model (SSM) was also performed on each set of bones using an open-source software (ShapeWorks v6.3.2, University of Utah; www.shapeworks.sci.utah.edu). SSM is a population-based mathematical approach to quantify morphological variation. While there are many uses for SSM, the two used for this study are calculating mean bone shapes and their associated correspondence particles. Fourteen SSMs were performed (one for each bone), and each bone was associated with one of the seven groups within a single bone optimized model. The overall mean shape of each bone was exported along with the group mean shape (Figure 5). In addition to the mean surfaces, the associated correspondence particles were exported. Correspondence particles are automatically placed using an entropy-based optimization scheme (Cates et al., 2017). Each particle is in the same relative location on the overall mean shape, for each of the group’s mean shapes and on the individual’s shapes.

#### 2.3.2 Automatic vs manual

**FIGURE 6:**
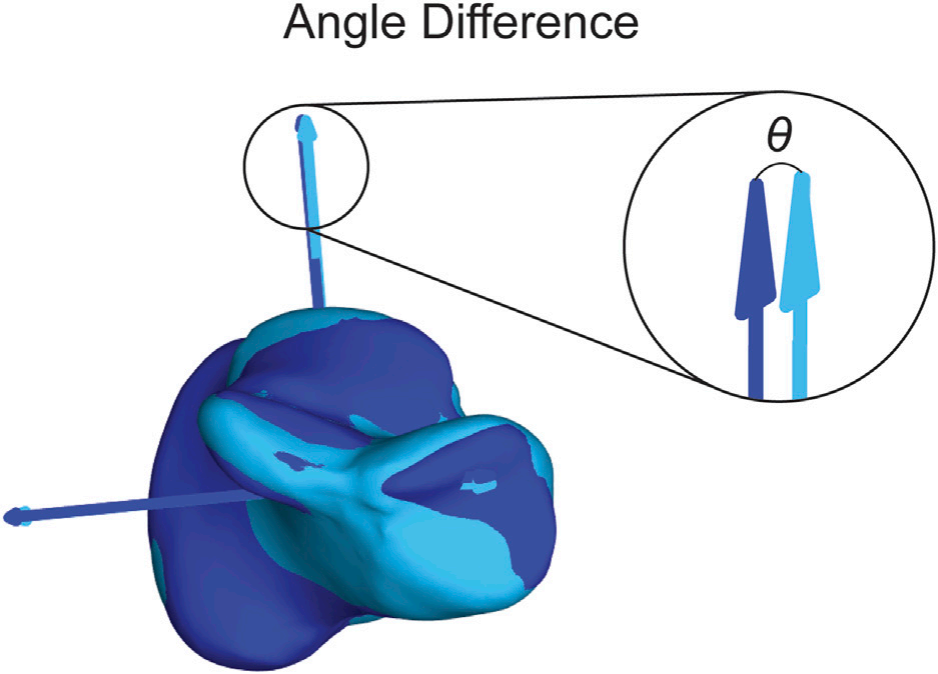
Visual representation of the angle difference calculation. The dark blue axes correlate to the dark blue bone and the light blue axes correlate to the light blue bone. The theta (θ) shown is the angle between the dark blue and light blue SI axes. The angle is also calculated between the dark blue and light blue ML and AP axes.

The first statistical analysis aimed to assess the consistency of the assigned ACSs when using different segmentation approaches. For each bone model, we assigned an ACS after automatic segmentation using DISIOR and after manual segmentation using Mimics. We will refer to these models as the Disior models and Mimics models, respectively. To quantify the variations between the ACSs obtained from the two segmentation approaches, we calculated the differences in angles on all three axes between the Disior model ACS and the Mimics model ACS (Figure 6). These differences in angles provide a measure of the disparity between the ACSs derived from the two segmentation methods. To determine the statistical significance of these differences across populations, a single-factor ANOVA analysis with a Tukey’s *post hoc* was performed on the angle difference data.

#### 2.3.3 Group mean vs individual

The second statistical analysis was used to determine how precise the assigned ACSs were within a group. We used the same ACS for the previously defined Mimics models and assigned ACSs to the group mean shape for each bone in each group. We then calculated the angled differences between the Mimics model ACSs and the group mean ACSs for each bone on all three axes (Figure 6). A single factor ANOVA analysis with a Tukey’s *post hoc* was performed on the angle difference data comparing across populations.

#### 2.3.4 Group mean vs overall mean

**FIGURE 7:**
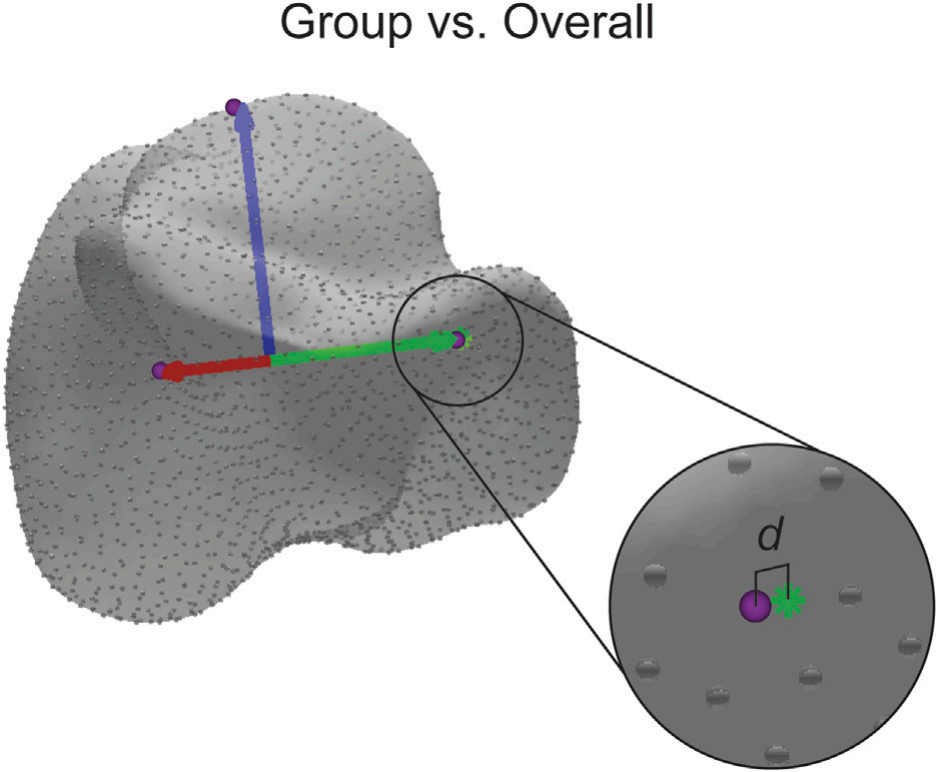
Visual representation of the distance difference calculation. The distance (d) reported is calculated between the identified correspondence particle (purple sphere) and the point where the axis intersects with the surface (green asterisk). The distance is also calculated in the same manner for the other axes.

The final statistical analysis was used to determine how accurate the assigned ACSs were across the seven population groups. For this study, accuracy is defined as the proportion of correct predictions among the total number of cases examined (i.e., the overall mean) assuming a Gaussian distribution (Allouche et al., 2006). In other words, accuracy is determined by how an examined individual groups falls relative to the Gaussian distribution of the entire population. First, the ACS of the overall mean shape, the average of all the groups combined, for each bone was calculated. For each axis, a point was projected along the axis to the surface of the bone. Using a nearest neighbor algorithm, the correspondence particle closest to the projected point was saved, and the Euclidean distance between the correspondence particle and the projected point was calculated. Then, the same process was performed on each of the group means, except the same correspondence particle number was used each time, and the Euclidean distance between the correspondence particle and the projected point was calculated (Figure 7). A single factor ANOVA analysis was performed on the distance difference data comparing across populations.

## 3 Results

Using AAFACT, the time taken to calculate a single ACS for a single bone is about 20 seconds, which includes the visual representation of the ACS overlaid on the bone model and a spreadsheet that contains the vectors of the ACS.

### 3.1 Automatic vs manual

Angle differences were calculated between the Disior model ACS and Mimics model ACS for each bone on all three axes. An average of 2.55° ± 1.94° was the angle difference for all bones, population groups, and axes. Specifically, the average asymptomatic angle difference was 2.01° ± 1.08°, the average OA angle difference was 2.77° ± 3.26°, the average pilon angle difference was 2.61° ± 1.77°, the average PCFD angle difference was 2.92° ± 2.14°, the average clubfoot angle difference was 2.45° ± 1.68°, the average CMT angle difference was 2.36° ± 1.49°, the average cavovarus angle difference was 2.71° ± 2.14°. There were no statistically significant differences between any of the groups. The overall mean values and the average values for each bone within each group is shown in Table 1.

**TABLE 1:** Angle differences ± standard deviation (SD) between the Disior model ACS and Mimics model ACS for each bone. An asterisk would indicate significant differences between groups, but no significant differences between groups were noted.

Automatic vs Manual (degrees)							
	Healthy	OA	PCFD	CMT	Pilon	Clubfoot	Cavovarus
CC Calcaneus	1.11 ± 0.49	3.12 ± 5.53	2.11 ± 2.06	1.32 ± 0.81	1.30 ± 1.03	1.44 ± 0.86	1.59 ± 0.89
ST Calcaneus	1.47 ± 0.97	3.67 ± 7.15	2.64 ± 1.97	3.05 ± 2.24	1.21 ± 0.73	2.62 ± 2.43	1.61 ± 0.70
TN Talus	2.23 ± 1.03	5.42 ± 8.20	3.49 ± 1.86	2.92 ± 1.36	2.37 ± 1.08	4.06 ± 3.82	2.52 ± 1.63
ST Talus	1.10 ± 0.60	2.38 ± 2.96	2.04 ± 0.97	1.48 ± 0.69	1.30 ± 0.74	2.06 ± 2.45	1.32 ± 0.79
TT Talus	2.59 ± 1.25	3.15 ± 2.67	3.99 ± 2.87	2.62 ± 1.53	2.58 ± 1.44	3.80 ± 1.04	2.36 ± 0.89
Navicular	1.50 ± 0.69	1.98 ± 1.69	1.26 ± 1.04	2.79 ± 1.32	1.49 ± 0.83	2.29 ± 1.47	2.39 ± 1.24
Cuboid	2.80 ± 1.84	4.66 ± 6.36	4.06 ± 2.07	3.51 ± 2.35	3.42 ± 1.94	4.50 ± 2.47	3.29 ± 1.88
Medial Cuneiform	2.12 ± 0.57	2.24 ± 2.27	3.06 ± 3.07	3.00 ± 2.17	2.41 ± 1.24	2.17 ± 0.99	4.85 ± 4.76
Intermediate Cuneiform	3.23 ± 1.11	2.19 ± 1.77	3.26 ± 1.95	3.31 ± 2.69	3.69 ± 1.38	3.48 ± 1.91	3.64 ± 2.40
Lateral Cuneiform	4.17 ± 2.48	3.98 ± 2.49	5.07 ± 3.10	3.93 ± 2.65	3.79 ± 3.18	4.27 ± 3.43	3.41 ± 2.91
Metatarsal 1	1.04 ± 0.63	1.21 ± 1.16	2.04 ± 1.77	1.14 ± 0.63	1.16 ± 0.39	0.74 ± 0.54	4.30 ± 5.56
Metatarsal 2	1.13 ± 0.63	1.31 ± 1.46	2.07 ± 1.21	1.00 ± 0.48	1.43 ± 1.04	1.06 ± 0.49	1.43 ± 1.13
Metatarsal 3	1.23 ± 0.58	1.80 ± 1.75	2.07 ± 1.91	1.40 ± 1.13	0.91 ± 0.67	0.73 ± 0.64	2.08 ± 1.70
Metatarsal 4	1.62 ± 1.43	1.37 ± 1.23	2.43 ± 3.33	1.38 ± 1.07	1.21 ± 0.76	0.86 ± 0.64	2.90 ± 2.84
Metatarsal 5	2.15 ± 1.12	1.84 ± 1.88	3.09 ± 2.67	1.62 ± 1.01	1.56 ± 1.05	2.28 ± 1.17	3.04 ± 1.68
Tibia	1.00 ± 0.61	1.46 ± 1.97	1.76 ± 1.46	1.93 ± 1.31	5.55 ± 2.91	1.41 ± 1.15	2.15 ± 3.63
Fibula	3.75 ± 2.34	5.38 ± 4.81	5.13 ± 3.09	3.75 ± 1.94	8.94 ± 9.61	3.90 ± 3.01	3.18 ± 1.68
Mean	2.01 ± 1.08	2.77 ± 3.26	2.92 ± 2.14	2.36 ± 1.49	2.61 ± 1.77	2.45 ± 1.68	2.71 ± 2.14

### 3.2 Group mean vs individual

Angle differences were calculated between the group mean ACS and individual Mimics model ACS for each bone on all three axes. An average of 2.72° ± 1.70° was the angle difference for all bones, population groups, and axes. Specifically, the average asymptomatic angle difference was 2.27° ± 1.31°, the average OA angle difference was 2.93° ± 1.83°, the average pilon angle difference was 2.96° ± 2.07°, the average PCFD angle difference was 2.52° ± 1.41°, the average clubfoot angle difference was 2.87° ± 2.09°, the average CMT angle difference was 2.91° ± 1.67°, the average cavovarus angle difference was 2.56° ± 1.55°. There were no statistically significant differences between any of the groups. The overall mean values and the average values for each bone within each group is shown in Table 2.

**TABLE 2:** Angle differences ± standard deviation (SD) between the group mean ACS and individual Mimics model ACS for each bone. An asterisk would indicate significant differences between groups, but no significant differences between groups were noted.

Mean vs Individual (degrees)							
	Healthy	OA	PCFD	CMT	Pilon	Clubfoot	Cavovarus
CC Calcaneus	3.12 ± 2.00	3.14 ± 1.71	2.21 ± 1.32	2.73 ± 1.60	2.71 ± 1.85	2.90 ± 2.30	2.90 ± 1.66
ST Calcaneus	3.10 ± 1.87	3.38 ± 2.15	3.71 ± 1.83	5.29 ± 2.67	3.51 ± 2.28	5.32 ± 6.50	3.66 ± 1.74
TN Talus	2.46 ± 1.15	3.66 ± 1.41	2.48 ± 1.25	3.77 ± 1.89	2.83 ± 1.80	2.39 ± 1.03	2.75 ± 1.57
ST Talus	1.21 ± 0.67	2.09 ± 0.80	1.29 ± 0.60	1.97 ± 1.13	1.39 ± 0.81	1.79 ± 1.10	1.20 ± 0.42
TT Talus	2.27 ± 1.33	4.22 ± 2.42	2.22 ± 1.30	3.46 ± 2.17	2.55 ± 1.15	2.98 ± 2.81	2.44 ± 1.15
Navicular	2.59 ± 1.21	3.10 ± 1.96	3.56 ± 1.77	3.87 ± 2.86	2.97 ± 1.70	2.71 ± 1.93	2.85 ± 1.78
Cuboid	3.65 ± 1.88	4.81 ± 2.51	4.15 ± 2.69	4.77 ± 3.05	4.29 ± 1.84	4.36 ± 2.71	4.66 ± 2.91
Medial Cuneiform	1.79 ± 1.09	1.77 ± 0.83	1.97 ± 0.82	3.34 ± 1.95	2.44 ± 1.63	2.75 ± 1.65	2.02 ± 1.67
Intermediate Cuneiform	2.92 ± 1.60	2.41 ± 1.45	2.07 ± 1.38	2.76 ± 1.21	2.85 ± 1.27	3.51 ± 1.98	2.07 ± 1.20
Lateral Cuneiform	2.59 ± 1.39	3.26 ± 2.11	3.08 ± 1.68	4.12 ± 1.64	2.85 ± 1.88	3.86 ± 2.22	2.38 ± 1.33
Metatarsal 1	1.19 ± 0.73	1.05 ± 0.80	1.07 ± 0.61	1.13 ± 0.54	1.17 ± 0.87	1.11 ± 0.53	1.13 ± 0.86
Metatarsal 2	1.93 ± 1.26	1.87 ± 1.47	1.99 ± 1.07	1.96 ± 1.08	2.21 ± 1.75	2.70 ± 1.63	2.09 ± 1.47
Metatarsal 3	1.76 ± 0.98	4.12 ± 4.59	1.20 ± 1.22	1.79 ± 1.39	1.52 ± 0.93	1.31 ± 0.78	1.17 ± 0.86
Metatarsal 4	1.25 ± 1.19	1.64 ± 1.18	1.29 ± 0.80	1.61 ± 1.03	1.91 ± 1.68	1.68 ± 1.57	2.35 ± 1.42
Metatarsal 5	2.67 ± 1.54	2.23 ± 1.30	2.20 ± 1.53	2.09 ± 1.34	2.54 ± 1.47	3.02 ± 1.34	2.63 ± 2.30
Tibia	1.98 ± 0.92	1.78 ± 0.88	2.68 ± 1.49	1.88 ± 0.94	4.73 ± 3.50	2.18 ± 2.40	2.09 ± 1.42
Fibula	2.16 ± 1.42	5.26 ± 3.51	5.67 ± 2.61	2.90 ± 1.87	7.77 ± 8.77	4.24 ± 3.01	5.15 ± 2.62
Mean	2.27 ± 1.13	2.93 ± 1.83	2.52 ± 1.41	2.91 ± 1.67	2.96 ± 2.07	2.87 ± 2.09	2.56 ± 1.55

### 3.3 Group mean vs overall mean

Distances were calculated between the identified correspondence particle and the projected point for each group on each bone and all three axes. An average of 0.21 mm ± 0.20 mm was the distance difference for all bones, population groups, and axes. Specifically, the average asymptomatic distance difference was 0.20 mm ± 0.15 mm, the average OA distance difference was 0.24 mm ± 0.27 mm, the average pilon distance difference was 0.15 mm ± 0.11 mm, the average PCFD distance difference was 0.25 mm ± 0.25 mm, the average clubfoot distance difference was 0.33 mm ± 0.36 mm, the average CMT distance difference was 0.16 mm ± 0.12 mm, the average cavovarus distance difference was 0.18 mm ± 0.18 mm. There were no statistically significant differences between any of the groups. The overall mean values and the average values for each bone within each group is shown in Table 3.

**TABLE 3:** Distances ±standard deviation (SD) between the identified correspondence particle and the projected point for each group on each bone. An asterisk would indicate significant differences between groups, but no significant differences between groups were noted.

Group vs Overall (mm)							
	Healthy	OA	PCFD	CMT	Pilon	Clubfoot	Cavovarus
CC Calcaneus	0.31 ± 0.35	0.49 ± 0.14	0.96 ± 0.79	0.24 ± 0.35	0.47 ± 0.48	1.00 ± 1.46	0.08 ± 0.07
ST Calcaneus	0.27 ± 0.20	0.07 ± 0.03	0.18 ± 0.28	0.21 ± 0.18	0.19 ± 0.16	0.18 ± 0.13	0.76 ± 0.72
TN Talus	0.23 ± 0.12	0.96 ± 0.79	0.09 ± 0.04	0.21 ± 0.15	0.18 ± 0.05	0.28 ± 0.27	0.39 ± 0.11
ST Talus	0.16 ± 0.10	0.42 ± 0.25	0.10 ± 0.02	0.16 ± 0.13	0.04 ± 0.04	0.42 ± 0.37	0.27 ± 0.04
TT Talus	0.08 ± 0.02	0.74 ± 0.57	0.21 ± 0.19	0.24 ± 0.10	0.27 ± 0.12	0.66 ± 0.51	0.21 ± 0.18
Navicular	0.22 ± 0.09	0.09 ± 0.08	0.22 ± 0.24	0.07 ± 0.06	0.13 ± 0.10	0.13 ± 0.04	0.12 ± 0.07
Cuboid	0.23 ± 0.05	0.22 ± 0.10	0.12 ± 0.14	0.23 ± 0.08	0.10 ± 0.04	0.08 ± 0.09	0.33 ± 0.15
Medial Cuneiform	0.10 ± 0.07	0.09 ± 0.06	0.30 ± 0.04	0.10 ± 0.09	0.07 ± 0.01	0.11 ± 0.14	0.11 ± 0.06
Intermediate Cuneiform	0.16 ± 0.05	0.16 ± 0.08	0.17 ± 0.05	0.13 ± 0.12	0.11 ± 0.04	0.17 ± 0.22	0.17 ± 0.19
Lateral Cuneiform	0.20 ± 0.11	0.20 ± 0.08	0.27 ± 0.15	0.03 ± 0.04	0.22 ± 0.13	0.30 ± 0.05	0.02 ± 0.01
Metatarsal 1	0.08 ± 0.06	0.06 ± 0.05	0.22 ± 0.11	0.12 ± 0.04	0.13 ± 0.10	0.16 ± 0.03	0.08 ± 0.07
Metatarsal 2	0.13 ± 0.19	0.10 ± 0.10	0.11 ± 0.13	0.09 ± 0.09	0.14 ± 0.18	0.13 ± 0.19	0.07 ± 0.08
Metatarsal 3	0.03 ± 0.03	0.03 ± 0.02	0.08 ± 0.04	0.12 ± 0.10	0.03 ± 0.01	0.03 ± 0.02	0.03 ± 0.03
Metatarsal 4	0.10 ± 0.03	0.12 ± 0.12	0.11 ± 0.09	0.08 ± 0.03	0.09 ± 0.07	0.08 ± 0.06	0.09 ± 0.05
Metatarsal 5	0.05 ± 0.05	0.06 ± 0.07	0.11 ± 0.07	0.06 ± 0.05	0.07 ± 0.05	0.23 ± 0.13	0.03 ± 0.03
Tibia	0.27 ± 0.05	0.10 ± 0.03	0.17 ± 0.04	0.15 ± 0.08	0.08 ± 0.04	0.31 ± 0.07	0.20 ± 0.09
Fibula	0.69 ± 0.49	0.10 ± 0.12	0.83 ± 0.59	0.54 ± 0.43	0.21 ± 0.13	1.34 ± 0.93	0.10 ± 0.06
Mean	0.20 ± 0.15	0.24 ± 0.27	0.25 ± 0.25	0.16 ± 0.12	0.15 ± 0.11	0.33 ± 0.36	0.18 ± 0.18

## 4 Discussion

In this study, we have presented a method and toolbox that allows for the automatic assignment of bone-specific and patient-specific ACSs for the 14 bones in the foot and ankle across seven different patient populations representing various deformities. Importantly, none of the coordinate systems generated by our toolbox are dependent on another bone. This means that researchers and practitioners have the flexibility to use the toolbox to analyze all bones together or focus on a single bone in isolation, without compromising the accuracy or reliability of the results. To assess the consistency, precision, and accuracy of the AAFACT across populations, we conducted three statistical analyses. Firstly, when evaluating the consistency of the toolbox, we compared the angle differences between the Disior model ACS and the Mimics model ACS, and our results revealed no significant differences between populations. Secondly, in assessing the precision of the toolbox, we compared the angle differences between the group mean ACS and the individual’s ACS, and again, no significant differences were observed across populations. Finally, to evaluate the accuracy of the toolbox, we calculated the distances between axes and the identified correspondence particle, and once more, no significant differences were found between populations.

These findings lead to several important conclusions. First, regarding the comparison between ACSs calculated from automatically segmented bones and manually segmented bones, the average angle differences were found to be less than 3°. However, even such slight variations may lead to subsequent kinematic errors (Long et al., 2008). Therefore, while the automatic segmentation processes are improving, it may still be beneficial to perform manual cleanup of segmentations to ensure higher accuracy to anatomically include bony features missed with automatic segmentation. It is important to note that manual segmentation can help mitigate potential errors that may arise from the current limitations of automatic segmentation methods. Nonetheless, when little to no manual segmentation is required, the automatically generated surfaces are sufficient for assigning an accurate ACS.

Second, in assessing the precision of the AAFACT, we observed larger variations between the group mean ACS and the individual ACS in cases of more severe pathologies such as the OA, pilon, and CMT groups. However, these variations were not statistically different from those observed in less severe pathologies. Therefore, the precision of the AAFACT was not significantly affected by the included morphological variations. This suggests that the toolbox can reliably capture a wide range of foot and ankle pathologies, regardless of their severity. Furthermore, the AAFACT demonstrated minimal variation between the mean ACSs of different pathological groups and the overall mean ACS. This indicates that the toolbox can precisely determine coordinates irrespective of the specific pathology. It effectively captures the unique morphology and alignment of individual bones, providing consistent and reliable ACS definitions.

It is worth noting that while techniques like Principal Component Analysis (PCA) can provide general representations of bone shapes, previous studies have shown that they do not consistently capture the varying morphological characteristics associated with different pathologies (Gutekunst et al., 2013; Lamm et al., 2016; Brown et al., 2020; Knutson et al., 2023). Additionally, approaches relying on landmark identification and geometric primitives may introduce human error during the manual selection process (Brown et al., 2020). The field currently lacks a widely accepted foot and ankle ACS standard based on modern imaging techniques and anatomical inconsistencies, leading to inconsistencies in comparing results across studies (Wu et al., 2002; Lenz et al., 2021). The AAFACT overcomes these limitations by automating the process and generating reliable, accurate, and precise ACSs that account for individual bone morphology and alignment.

When using ACSs, it is essential to consider the distinct functional roles and articulations of these bones within the foot and ankle complex. The foot and ankle consist of multiple joints that work together to enable complex movements and weightbearing functions. For instance, the talus is a key component in tibiotalar dorsiflexion and plantarflexion, while the subtalar joint is partially responsible for pronation and supination movements (Jastifer and Gustafson, 2014). By defining separate joint coordinate systems for these specific bones, the AAFACT aims to capture the unique kinematics and functional contributions of each joint, allowing for a comprehensive analysis of foot and ankle dynamics in future work.

Additionally, the AAFACT has been designed to accommodate partial and full-length bones, thereby increasing its versatility for analyzing data from various WBCT devices. In this study, we focused on including both partial tibias and fibulas and full-length metatarsals obtained from multiple different WBCT scans with varying cropped fields of view. This design allows us to address scenarios where only partial bone models are available, which can arise from using WBCT scans acquired with different imaging protocols or devices featuring smaller field of view capabilities. By incorporating these partial bone models, the AAFACT demonstrates its adaptability to varying imaging setups, enhancing its applicability across WBCT modalities and devices. Built in capability also provides a warning if cropped bones are too limited which would contribute to excessive errors in the ACS identification (Muhlrad et al., 2022).

This study has limitations. First, there is a limited number of studies to compare, further emphasizing the need for ACSs to be vetted across multiple pathologies. Second, because the analysis used high resolution scans to determine reliability, there is no guarantee that low resolution scans will have the same level reliability. Additionally, these ACS should be analyzed via dynamic movement trials to evaluate planar kinematics to have complete confidence that the AAFACT is minimizing kinematic crosstalk in joint angle calculations. Future work includes utilizing the toolbox to study dynamic data, analyzing joint angle variations, and determining if the ACS presents any crosstalk during dynamic activities.

A precise and reliable ACS is crucial for gaining a comprehensive understanding of bone position and orientation in the foot and ankle across diverse populations. By accurately defining ACSs, clinicians and engineers can obtain precise measurements and assessments of bone morphology, alignment, and joint kinematics. This information is invaluable for clinical decision-making, surgical planning, implant design, and biomechanical analysis.

The automatic calculation of ACSs through the AAFACT offers significant advantages for both clinicians and engineers. Clinicians can benefit from a standardized and objective method for assessing foot and ankle pathologies, enabling more accurate diagnoses and personalized treatment strategies. Engineers can utilize precise ACS data to develop and refine biomechanical models, simulate joint movements, and optimize orthopedic devices. The AAFACT bridges the gap between clinical practice and engineering applications, providing a practical and efficient tool for multidisciplinary collaboration.

In conclusion, the development of the open-source AAFACT and the automatic assignment of ACSs contribute significantly to the field of foot and ankle research by for a standardized and widely accepted anatomical coordinate system. By accurately defining and analyzing ACSs, this toolbox empowers clinicians and engineers with a tool for studying foot and ankle pathologies, improving treatment outcomes, and advancing our understanding of the complex biomechanics of the lower extremities.

## Data Availability

The original contributions presented in the study are included in the article/Supplementary Material, further inquiries can be directed to the corresponding author.
